# Evaluation of Candidate Urinary microRNAs in Upper Tract Urothelial Carcinoma: A Prospective Multicenter Biomarker Study (JCOG1403A1)

**DOI:** 10.3390/genes17091145

**Published:** 2026-09-18

**Authors:** Shuichi Tatarano, Hideki Enokida, Hiroyuki Nishiyama, Takahiro Kojima, Hirofumi Yoshino, Takahiko Mitsui, Akihiro Ito, Tomonori Habuchi, Keisuke Kanato, Hiroshi Kitamura

**Affiliations:** 1 Department of Urology, Graduate School of Medical and Dental Sciences, Kagoshima University, Kagoshima 890-8544, Kagoshima, Japan; henokida@m2.kufm.kagoshima-u.ac.jp (H.E.); hyoshino@m3.kufm.kagoshima-u.ac.jp (H.Y.); 2 Department of Urology, Institute of Medicine, University of Tsukuba, Tsukuba 305-8575, Ibaraki, Japan; nishiuro@md.tsukuba.ac.jp; 3 Department of Urology, Aichi Cancer Center, Nagoya 464-8681, Aichi, Japan; t.kojima@aichi-cc.jp; 4 Department of Urology, Graduate School of Medical Sciences, University of Yamanashi, Chuo 409-3898, Yamanashi, Japan; tmitsui@yamanashi.ac.jp; 5 Department of Urology, Graduate School of Medicine, Tohoku University, Sendai 980-8574, Miyagi, Japan; akihiro.ito.c5@tohoku.ac.jp; 6 Department of Urology, Graduate School of Medicine, Akita University, Akita 010-8543, Akita, Japan; thabuchi@doc.med.akita-u.ac.jp; 7 JCOG Data Center/Operating Office, National Cancer Center Hospital, Chuo-ku 104-0045, Tokyo, Japan; keisuke-kanato@amed.go.jp; 8 Department of Urology, University of Toyama, Toyama 930-0194, Toyama, Japan; hkitamur@med.u-toyama.ac.jp

**Keywords:** upper tract urothelial carcinoma, microRNA, urinary biomarker, urine cytology, relapse-free survival

## Abstract

Objectives: To evaluate the clinical associations of candidate urinary microRNAs (miRNAs) in patients with upper tract urothelial carcinoma (UTUC), including their ability to discriminate patients with UTUC from healthy controls and their associations with relapse-free survival (RFS). Methods: This prospective multicenter ancillary study of the JCOG1403 trial (JCOG1403A1 study) included 45 patients with UTUC and 16 healthy controls. Four candidate miRNAs (miR-130b, miR-182, miR-191, and miR-200a) were selected based on prior exploratory sequencing analysis. Urinary cell fractions were isolated by cell sorting, and miRNA expression was quantified by stem-loop RT-qPCR. The ability of urinary miRNAs to discriminate patients with UTUC from healthy controls, their associations with clinicopathological characteristics, and their associations with RFS were evaluated. Results: Among the candidate miRNAs, miR-191 and miR-200a showed significantly increased expression in patients with UTUC and demonstrated favorable discrimination between patients with UTUC and healthy controls. The combined miR-191/miR-200a model showed the highest discriminatory performance (area under the curve, 0.888). Urine cytology was positive in 33.3% of evaluable patients, whereas urinary miR-191 and miR-200a were detectable in 78.6% and 85.7% of patients with negative/suspicious cytology, respectively. Preoperative urinary miR-182 and miR-191 detectability was significantly associated with pathological tumor stage. In exploratory prognostic analyses, patients with detectable postoperative urinary miR-130b had significantly shorter RFS (log-rank *p* = 0.0455)**;** however, the association was not statistically significant in multivariable Cox regression analysis (hazard ratio 2.76, 95% confidence interval 0.84–9.05, *p* = 0.094). Conclusions: Urinary miR-191 and miR-200a showed potential for discriminating patients with UTUC from healthy controls and may complement urine cytology, particularly in patients with negative/suspicious cytology. The association between postoperative urinary miR-130b detectability and RFS should be considered exploratory and requires further validation.

## 1. Introduction

Upper tract urothelial carcinoma (UTUC) is a relatively uncommon malignancy, accounting for approximately 5–10% of all urothelial carcinomas [1]. Despite advances in imaging modalities and endoscopic techniques, the diagnosis of UTUC remains challenging because of its anatomical location and the limited sensitivity of currently available urinary biomarkers [2].

Urine cytology is widely used as a noninvasive diagnostic tool for urothelial carcinoma. However, its sensitivity is suboptimal, particularly for low-grade tumors and upper urinary tract lesions [2]. Although several urinary biomarkers have been investigated for bladder cancer, reliable biomarkers for the detection and monitoring of UTUC have not yet been established. Therefore, the development of novel noninvasive biomarkers for UTUC remains an unmet clinical need.

MicroRNAs (miRNAs) are small noncoding RNAs that regulate gene expression and play important roles in cancer development and progression [3]. Owing to their remarkable stability in biological fluids, urinary miRNAs have attracted considerable interest as potential biomarkers for urothelial malignancies [4]. Several studies have reported associations between specific miRNAs and the diagnosis, progression, or prognosis of urothelial carcinoma [5,6,7,8,9,10,11,12]. However, most previous investigations have focused on bladder cancer, and evidence regarding urinary miRNAs in UTUC remains limited because of the rarity of the disease and the difficulty in obtaining adequately sized cohorts [13,14,15,16]. Consequently, validation studies using prospectively collected urine samples from well-characterized UTUC cohorts remain scarce.

Based on a prior exploratory sequencing analysis performed by our institution and previous biological evidence, four candidate miRNAs (miR-130b-3p, miR-182-5p, miR-191-5p, and miR-200a-3p) were selected for further validation (Supplementary Table S1).

In the present ancillary study of the prospective multicenter JCOG1403 trial [17], we evaluated the discriminatory performance of four candidate urinary miRNAs and urinary sorting cell counts for distinguishing patients with UTUC from healthy controls. We further assessed their associations with urine cytology, clinicopathological characteristics, and relapse-free survival (RFS) after radical nephroureterectomy to explore their potential clinical relevance in UTUC.

## 2. Materials and Methods

### 2.1. Study Design and Patients

This study was conducted as part of JCOG1403A1, a prospective multicenter biomarker study ancillary to the JCOG1403 study, a randomized phase III trial conducted at 43 institutions across Japan. The parent JCOG1403 study evaluated the efficacy and safety of a single early intravesical instillation of pirarubicin for preventing bladder recurrence after radical nephroureterectomy in patients with UTUC. The detailed study protocol has been published previously [17]. According to the JCOG1403 protocol, RFS was defined as the time from the second registration (randomization) to the earliest occurrence of relapse, including intravesical relapse, or death from any cause, whichever occurred first. Patients without an event were censored at the date of last follow-up. JCOG1403A1 was designed to evaluate several candidate biomarkers using prospectively collected biospecimens from patients with UTUC. The present study represents one of the predefined biomarker analyses within JCOG1403A1 and evaluated the ability of candidate urinary miRNAs to discriminate patients with UTUC from healthy controls, their associations with clinicopathological characteristics, and their associations with clinical outcomes.

Eligible patients were those with clinically localized UTUC staged as cTa–cT3N0M0 based on computed tomography findings. Additional key inclusion criteria included no prior history of UTUC or bladder cancer, an Eastern Cooperative Oncology Group performance status of 0–1, and adequate organ function.

**Figure 1:**
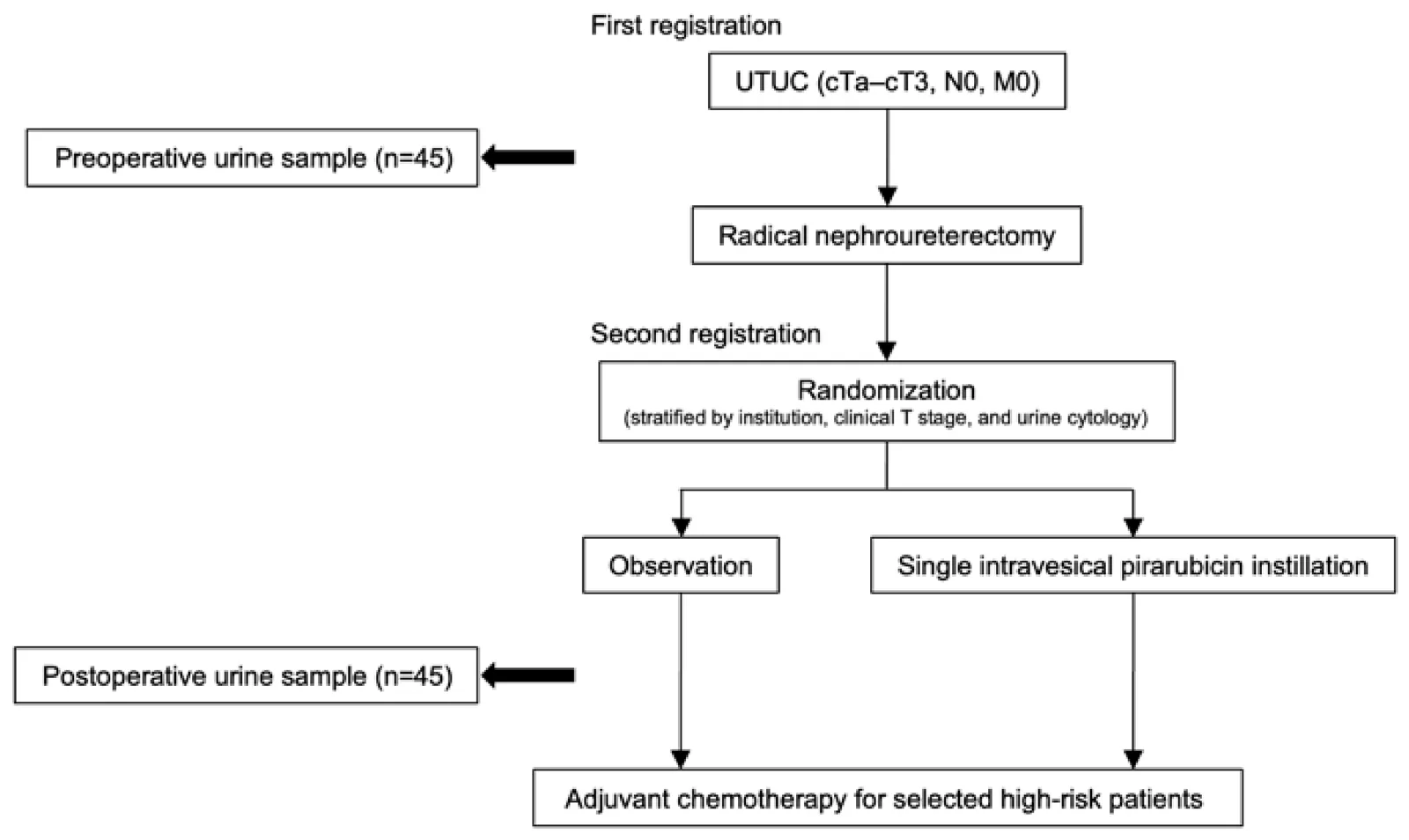
Study design and urine sample collection schedule. Forty-five patients with clinically localized UTUC (cTa–cT3N0M0) enrolled in JCOG1403 provided urine samples before and after radical nephroureterectomy. Following surgery, patients underwent a second registration and were randomized to observation or a single intravesical pirarubicin instillation. Postoperative urine samples were also collected before randomization. Selected high-risk patients received adjuvant chemotherapy according to the study protocol. Black arrows indicate the timing of urine sample collection.

After radical nephroureterectomy, eligible patients underwent the second registration and were randomly assigned to either the pirarubicin instillation arm or the observation arm. Pathological staging was performed according to the Union for International Cancer Control TNM Classification of Malignant Tumors, 7th edition, and tumor grade was classified according to the 2004 World Health Organization/International Society of Urological Pathology classification. Divergent differentiation included squamous and glandular differentiation identified in pathological specimens. The study workflow and urine sample collection schedule are shown in Figure 1. For comparison with patients with UTUC, urine samples were obtained from healthy controls who were physicians and had provided broad consent for the use of their specimens in research at the institution where the miRNA analyses were performed.

The study was conducted in accordance with the Declaration of Helsinki and the Ethical Guidelines for Medical and Biological Research Involving Human Subjects in Japan. The JCOG1403A1 protocol was reviewed and approved by the JCOG Protocol Review Committee and subsequently approved by the institutional review board or ethics committee at each participating institution. Written informed consent and the use of existing specimens under an opt-out procedure were implemented in accordance with the approved study protocol and the applicable ethical guidelines in Japan.

### 2.2. Urine Sample Collection, Processing, and Cytology

Urine samples (50 mL each) were collected before and after radical nephroureterectomy at each participating institution, and sent to SRL Inc. (Tokyo, Japan). SRL Inc. then forwarded the samples to Kagoshima University, the facility responsible for analysis. Urine samples were centrifuged to collect the cell pellet fraction. The obtained pellets were resuspended and preserved in a 30% polyethylene glycol (PEG) solution. This solution was prepared by dissolving PEG 8000 (Sigma-Aldrich, St. Louis, MO, USA) in 3M NaCl to a final concentration of 30% (*w*/*v*).

Urine cytology was assessed preoperatively at each participating institution using naturally voided urine, without central cytological review. The JCOG1403 protocol did not specify the professional category of the cytology assessor or mandate a uniform cytomorphological classification system or morphological criteria for cytological positivity across participating institutions. According to the protocol, one cytology result was sufficient when positive, whereas three results were required when negative or suspicious; once a positive result was obtained, no further cytological examination was required. For the present analysis, cytology results were categorized as positive or negative/suspicious based on the results recorded in the JCOG1403 study. Patients with unavailable cytology results were excluded from analyses involving urine cytology.

### 2.3. Selection of Candidate miRNAs

Candidate miRNAs were selected based on prior exploratory sequencing analysis using urinary samples from three patients with UTUC and three healthy controls. The exploratory miRNA sequencing analysis was performed by RIKEN GENESIS CORPORATION (Tokyo, Japan), and differential expression analysis between the UTUC and healthy control groups was performed using the edgeR version 3.8.6 with a generalized linear model (GLM). Group comparisons were performed using the likelihood ratio test implemented in the glmLRT function. The miRNAs identified in the exploratory sequencing analysis were ranked according to a composite expression score generated from the sequencing data, which incorporated expression difference, fold change, variability, and statistical significance between the UTUC and healthy control groups. The expression score was calculated as follows: Score = (Mean Cancer − Mean Normal) × logFC/(SD Cancer + SD Normal)/*p* value, where SD represents the population standard deviation. The logFC values were obtained from the differential expression analysis using edgeR, and logFC was not calculated directly from the ratio of the group mean expression values. Group-level expression data and the components used to calculate the expression score are provided in Supplementary Table S2 Part A. The four candidate miRNAs (miR-130b, miR-182, miR-191, and miR-200a) were selected by considering both the results of the exploratory sequencing analysis and previous evidence supporting their potential relevance to urothelial carcinoma, rather than solely on the basis of the expression-score ranking. The characteristics of the selected miRNAs and previous evidence regarding their relevance to urothelial carcinoma are summarized in Supplementary Table S1. Individual sample-level expression data from the exploratory sequencing analysis are provided in Supplementary Table S2 Part B.

### 2.4. Fluorescence-Activated Cell Sorting (FACS)

Cellular components were analyzed and sorted using a BD FACSAria II cell sorter (BD Biosciences, San Jose, CA, USA). Urinary cell pellets preserved in 30% polyethylene glycol (PEG) were subjected directly to cell sorting without additional washing or buffer exchange. A urinary cell fraction was isolated by excluding hematological cells based on physical properties, including cell size (forward scatter) and internal complexity/granularity (side scatter). The gating strategy was established in preliminary experiments using mixtures of blood cells and GFP-expressing T24 urothelial carcinoma cells, based on differences in their scatter characteristics. Preliminary experiments confirmed that preservation in 30% PEG maintained RNA quality and allowed subsequent cell sorting. Because no urothelial-specific marker was used, the cellular composition and purity of the isolated fraction were not directly validated. The isolated fraction is therefore referred to as the “FACS-selected urinary cell fraction” in the present study. The FACS-selected urinary cell fraction was subsequently collected for molecular analysis. The number of cells recovered in this fraction from each urine sample was recorded as the urinary sorting cell count and used for subsequent analyses. This cell-sorting methodology is protected under Japanese Patent No. 6898630 [18].

### 2.5. RNA Extraction and Stem-Loop RT-qPCR for miRNA Quantification

Total RNA was extracted from the FACS-selected urinary cell fractions using the phenol–chloroform method with ISOGEN (Nippon Gene Co., Ltd., Tokyo, Japan) according to the manufacturer’s instructions. The quality and integrity of the extracted RNA were assessed using an RNA 6000 Nano Assay Kit and a 2100 Bioanalyzer (Agilent Technologies, Santa Clara, CA, USA). For miRNA expression analysis, stem-loop reverse transcription-quantitative polymerase chain reaction (RT-qPCR) was performed. Complementary DNA was synthesized from total RNA using miRNA-specific primers from the TaqMan MicroRNA Assay (Thermo Fisher Scientific, Waltham, MA, USA). Quantitative PCR was then conducted using an ABI 7300 Sequence Detection System (Thermo Fisher Scientific). Because the concentration of total RNA extracted from urinary cell fractions was too low for accurate quantification in some samples, a fixed volume (5 μL) of total RNA was used for reverse transcription, as previously described [10]. Consistent with our previously reported approach for urinary miRNA analysis, no endogenous reference RNA was used for normalisation. Because the measured urinary miRNAs may originate not only from cells within the FACS-selected urinary cell fraction but also from fluid components associated with these cells, normalisation to an intracellular endogenous reference may not appropriately account for miRNAs present in the cell-associated fluid fraction. Therefore, consistent with our previously reported approach for urinary miRNA analysis, target miRNA levels were evaluated using raw Ct values without normalisation to an endogenous internal reference. This approach was supported by our previous validation demonstrating a strong linear relationship between the amount of target miRNA and the corresponding Ct value using serial dilutions of known amounts of miRNA [10]. The quantitative relationship between miRNA input and Ct value has also been demonstrated in circulating miRNA assays using serial dilutions of synthetic miRNA [19]. Samples with Ct values < 45 were defined as detectable, whereas those with Ct values ≥ 45 were considered nondetectable. For samples in which amplification was not detected, a Ct value of 45 was assigned and used for subsequent statistical analyses, including receiver operating characteristic (ROC) curve analysis. For graphical visualization only, Ct values were transformed as 2^^(45−Ct)^, hereafter referred to as the Ct-transformed signal, with a Ct value of 45 corresponding to a Ct-transformed signal of 1. All statistical comparisons and ROC analyses were performed using the Ct values rather than Ct-transformed signal.

### 2.6. Statistical Analyses

Preoperative urine samples were used for analyses of urinary sorting cell counts, miRNA expression, discriminatory performance, associations with urine cytology, and clinicopathological characteristics, whereas postoperative urine samples were used for prognostic analyses of the association between miRNA detectability and RFS.

Differences in miRNA expression levels between groups were analyzed using the Mann–Whitney U test. Correlations between urinary sorting cell counts and miRNA expression levels were assessed using Spearman’s rank correlation coefficient. Associations between categorical variables were evaluated using Fisher’s exact test. The diagnostic yield of urinary miRNAs and urine cytology was compared using McNemar’s test.

ROC curve analysis was performed to evaluate the discriminatory performance of urinary sorting cell counts and candidate miRNAs for distinguishing patients with UTUC from healthy controls. A combined model incorporating miR-191 and miR-200a Ct values was constructed using logistic regression, and its discriminatory performance was evaluated using ROC curve analysis. Exploratory cut-off values were determined using the Youden index. For the primary ROC analyses in the overall cohort, AUCs are reported with 95% confidence intervals (CIs) estimated using bias-corrected bootstrap resampling with 5000 replicates. Sensitivity and specificity at the exploratory cut-off values are reported with exact binomial (Clopper–Pearson) 95% CIs. To assess whether the discrimination between patients with UTUC and healthy controls based on miRNA levels was independent of sorted cell count, multivariable logistic regression analyses were performed using miR-191 or miR-200a Ct values and log10(sorted cell count + 1) as explanatory variables. To assess the potential influence of differences in age and sex distribution between patients with UTUC and healthy controls, a sensitivity analysis restricted to male participants was performed for preoperative miR-191 and miR-200a Ct values using the Mann–Whitney U test and ROC analysis. Correlations between age and preoperative miR-191 and miR-200a Ct values within the UTUC cohort were assessed using Spearman’s rank correlation coefficient.

RFS was estimated using the Kaplan–Meier method and compared using the log-rank test. Univariate and multivariable Cox proportional hazards regression analyses were performed to identify factors associated with RFS.

All statistical analyses were performed using JMP Pro version 19 software (SAS Institute Inc., Cary, NC, USA). Two-sided *p* values < 0.05 were considered statistically significant.

## 3. Results

### 3.1. Patient Characteristics

A total of 45 patients with UTUC and 16 healthy controls were included in this study. Detailed demographic and clinicopathological characteristics are summarized in Table 1. Among the patients with UTUC, the median age was 71 years (range, 37–83 years), and 31 patients (69%) were male. Most tumors were high grade (78%) and pathologically invasive (≥pT2, 56%). Concomitant carcinoma in situ was present in 18 patients (40%), and lymphovascular invasion was identified in 8 patients (18%).

### 3.2. Preoperative Urinary Sorting Cell Counts and Expression Levels of Candidate miRNAs

**Figure 2:**
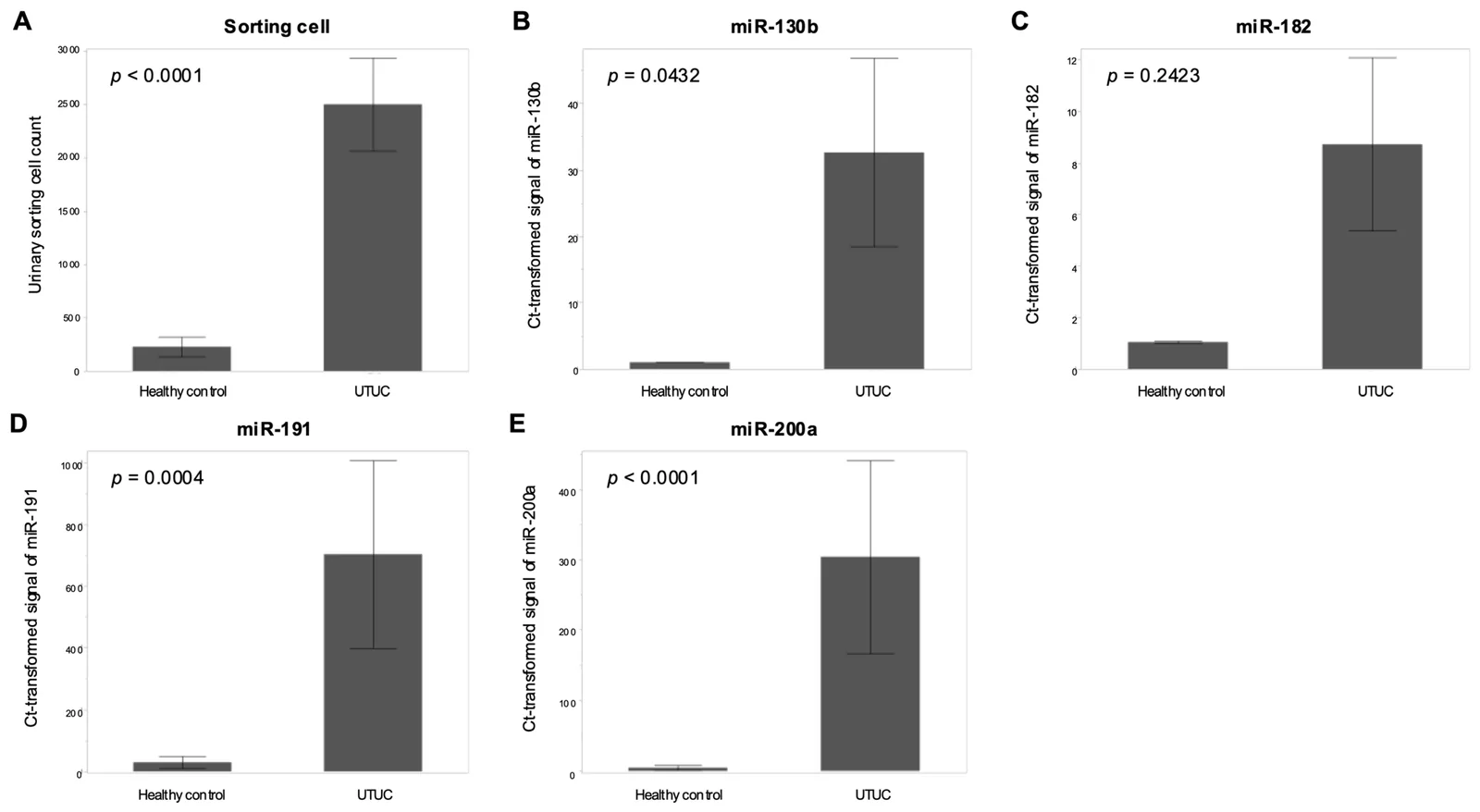
Urinary sorting cell counts and expression levels of candidate miRNAs in healthy controls and patients with UTUC. (**A**) Urinary sorting cell counts. (**B**–**E**) Ct-transformed signals of miR-130b, miR-182, miR-191, and miR-200a. Ct-transformed signals were calculated as 2^^(45−Ct)^. Bars indicate mean ± SEM. *p* values were calculated using the Mann–Whitney U test.

In preoperative urine samples, urinary sorting cell counts were significantly higher in patients with UTUC than in healthy controls (Figure 2A). Among the four candidate miRNAs evaluated, miR-191 (Figure 2D) and miR-200a (Figure 2E) showed significantly higher Ct-transformed signals in patients with UTUC than in healthy controls (both *p* < 0.001), whereas miR-130b showed a modest but significant difference (Figure 2B; *p* = 0.0432). No significant difference was observed for miR-182 (Figure 2C; *p* = 0.2423).

To assess the potential influence of sorted cell count on the observed differences in miRNA levels, additional multivariable logistic regression analyses were performed. After adjustment for log10(sorted cell count + 1), both miR-191 Ct (OR, 0.598; 95% CI, 0.373–0.809; *p* = 0.008) and miR-200a Ct (OR, 0.433; 95% CI, 0.204–0.684; *p* = 0.005) remained significantly associated with UTUC (Supplementary Table S5).

To assess the potential influence of the sex imbalance between the groups, a sensitivity analysis was performed restricted to male participants (31 patients with UTUC and 16 healthy controls). Significant differences between patients with UTUC and healthy controls were retained for both miR-191 (*p* = 0.0014; AUC, 0.770) and miR-200a (*p* < 0.0001; AUC, 0.862) (Supplementary Table S6). As an exploratory assessment of the potential influence of age, correlations between age and miRNA Ct values were examined within the UTUC cohort. Age was not significantly correlated with miR-191 Ct (Spearman’s ρ = −0.158, *p* = 0.299) or miR-200a Ct (ρ = −0.204, *p* = 0.179) (Supplementary Table S6).

### 3.3. Discriminatory Performance of Preoperative Urinary Sorting Cell Counts and Candidate miRNAs

**Figure 3:**
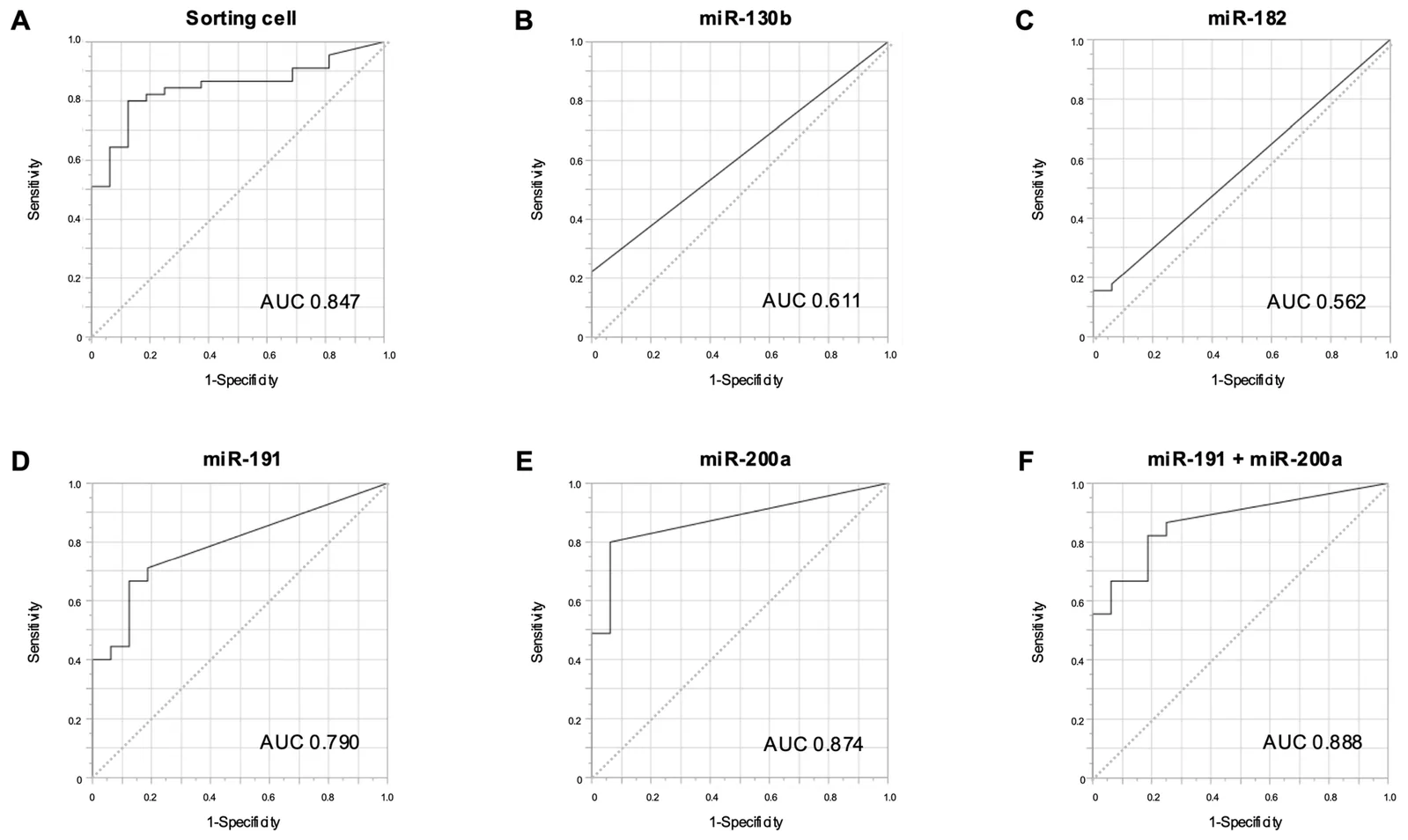
Discriminatory performance of preoperative urinary sorting cell counts and candidate miRNAs for distinguishing patients with UTUC from healthy controls. Receiver operating characteristic (ROC) curve for (**A**) urinary sorting cell counts, (**B**) miR-130b, (**C**) miR-182, (**D**) miR-191, (**E**) miR-200a, and (**F**) the combined miR-191/miR-200a model. For individual miRNAs, samples in which the respective miRNA was undetectable were assigned a Ct value of 45 for ROC analysis. Areas under the curve (AUCs) are shown. The dashed diagonal line represents the reference line corresponding to an AUC of 0.5.

ROC curve analysis demonstrated that urinary sorting cell counts achieved an area under the curve (AUC) of 0.847 for distinguishing patients with UTUC from healthy controls. Among the candidate miRNAs, miR-200a showed the highest discriminatory performance (AUC, 0.874; 95% CI, 0.780–0.940), followed by miR-191 (AUC, 0.790; 95% CI, 0.674–0.881). In contrast, miR-130b and miR-182 were detectable in only 10 of 45 (22.2%) and 8 of 45 (17.8%) patients with UTUC, respectively, and showed limited discriminatory performance (AUC, 0.611 and 0.562, respectively) (Figure 3, Table 2). The combined miR-191/miR-200a model yielded an AUC of 0.888 (95% CI, 0.778–0.948). At the exploratory cut-off determined using the Youden index, the combined model showed a sensitivity of 73.3% and a specificity of 93.8%. Detailed discriminatory performance, including 95% CIs for sensitivity and specificity, is provided in Supplementary Table S7.

### 3.4. Comparison of miRNA Detectability with Urinary Cytology

Preoperative urine cytology results were available for 42 of the 45 patients with UTUC. Among these evaluable patients, urine cytology was positive in 14 (33.3%). Detection rates were 71.1% for miR-191 and 80.0% for miR-200a (Table 3). Among patients with negative/suspicious cytology (n = 28), urinary miR-191 and miR-200a were detectable in 22 (78.6%) and 24 (85.7%) patients, respectively. When detectability of either miR-191 or miR-200a was considered, at least one of these miRNAs was detectable in 25 of 28 (89.3%) patients with negative/suspicious cytology (Supplementary Table S3).

### 3.5. Association Between Preoperative Urinary miRNA Detectability and Clinicopathological Features

The detectability of preoperative urinary miR-182 and miR-191 was significantly associated with pathological tumor stage. Detectable miR-182 was observed in 32.0% of patients with ≥pT2 disease but in none of those with <pT2 disease (*p* = 0.0056). Similarly, miR-191 detectability was significantly higher in patients with ≥pT2 disease than in those with <pT2 disease (84.0% vs. 55.0%, *p* = 0.0488). No significant associations were observed for miR-130b or miR-200a (Table 4).

### 3.6. Correlations Between Preoperative Urinary Sorting Cell Counts and miRNA Expression

In the overall cohort, urinary sorting cell counts were significantly correlated with expression levels of miR-130b (ρ = −0.3317, *p* = 0.0090), miR-191 (ρ = −0.4330, *p* = 0.0005), and miR-200a (ρ = −0.4391, *p* = 0.0004). No significant correlation was observed between sorting cell counts and miR-182 expression (ρ = −0.1341, *p* = 0.3027) (Supplementary Figure S1A–D).

### 3.7. Association Between Postoperative Urinary miRNA Detectability and RFS

**Figure 4:**
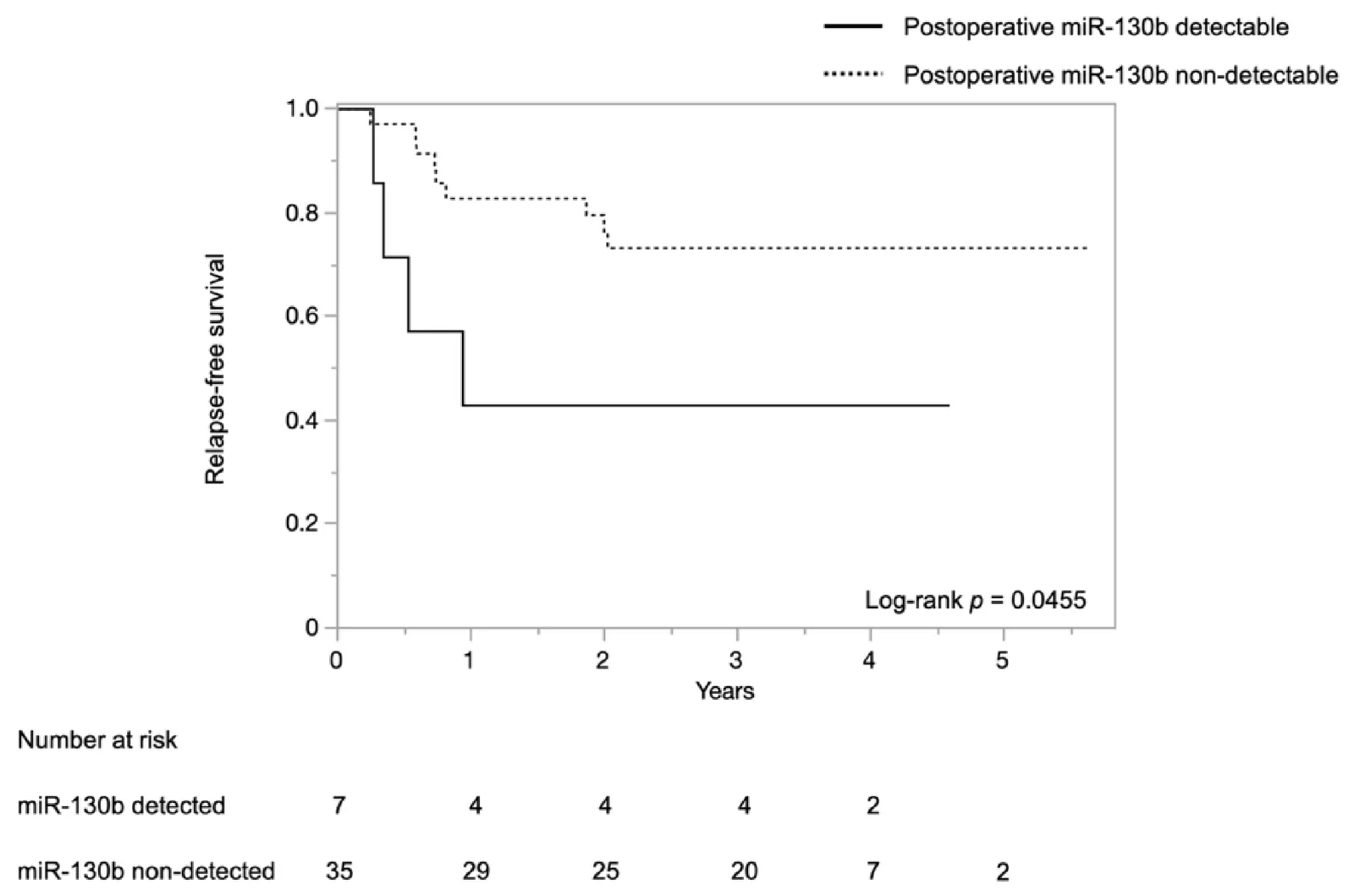
Relapse-free survival according to postoperative urinary miR-130b detectability. Kaplan–Meier curves for relapse-free survival stratified by postoperative urinary miR-130b detectability. Detectability was defined as Ct < 45. *p* values were calculated using the log-rank test.

Among the 45 patients with postoperative urine samples, RFS data were available for 42 patients, who were included in the prognostic analyses. In an exploratory Kaplan–Meier analysis, patients with detectable postoperative urinary miR-130b showed shorter RFS than those without detectable miR-130b (log-rank *p* = 0.0455; Figure 4). No significant associations with RFS were observed for postoperative miR-182, miR-191, or miR-200a detectability (Supplementary Figure S2A–C).

In univariate Cox proportional hazards analysis, postoperative miR-130b detectability was associated with an HR of 3.14 (95% CI, 0.96–10.25; *p* = 0.058), although the association did not reach statistical significance (Table 5 and Supplementary Table S4). Similarly, after adjustment for postoperative intravesical pirarubicin instillation, the association remained non-significant (HR, 2.76; 95% CI, 0.84–9.05; *p* = 0.094) (Table 5).

## 4. Discussion

In this ancillary analysis of the prospective multicenter JCOG1403 trial, we evaluated four candidate urinary miRNAs using prospectively collected preoperative and postoperative urine samples from patients with UTUC. The principal findings were as follows: (i) miR-191 and miR-200a showed favorable discrimination between patients with UTUC and healthy controls and were frequently detectable in patients with negative/suspicious urine cytology; (ii) preoperative miR-182 and miR-191 detectability was associated with pathological tumor stage; and (iii) postoperative miR-130b detectability showed an exploratory association with RFS. These findings provide a basis for further investigation of urinary miRNAs in UTUC, while the exploratory nature of the study and the characteristics of the control group warrant cautious interpretation.

Among the four candidate miRNAs, miR-191 and miR-200a showed the most favorable discrimination between patients with UTUC and healthy controls. Previous studies have reported dysregulation of these miRNAs in urothelial carcinoma. In a prospective study of urinary cell-free miRNAs in bladder cancer, Juracek et al. identified increased urinary miR-191-5p levels in patients with bladder cancer compared with healthy controls [20], while experimental studies have demonstrated overexpression of miR-200a in bladder cancer tissues and a potential role in tumor invasion [21]. Although relatively few studies have specifically investigated urinary miRNAs in UTUC, Karanović et al. identified UTUC-associated urinary miRNA profiles and demonstrated changes in urinary miRNA expression before and after tumor resection [16]. More recently, a prospective multicenter study demonstrated favorable discriminatory performance of an eight-gene urinary RNA panel for UTUC [22]. Furthermore, a recent systematic review by the EAU Guidelines UTUC Panel identified RNA-based urinary assays among several promising liquid-based biomarker approaches for UTUC, while emphasizing the limited certainty of the available evidence and the need for prospective validation [23]. These observations support continued investigation of urinary RNA-based biomarkers in UTUC.

An important methodological consideration is the potential influence of urinary cell yield on miRNA measurements. Urinary sorting cell counts were higher in patients with UTUC than in healthy controls and were correlated with the Ct values of several candidate miRNAs, raising the possibility that differences in recovered cell numbers contributed to the observed miRNA differences. However, after adjustment for sorted cell count, both miR-191 and miR-200a Ct values remained significantly associated with UTUC status. These findings suggest that the differences in miR-191 and miR-200a between patients with UTUC and healthy controls were not solely attributable to differences in cellular yield. Nevertheless, because a fixed volume rather than an equal amount of total RNA was used for reverse transcription, the potential influence of differences in cellular yield cannot be completely excluded.

The relationship between urinary miRNA detectability and urine cytology may also be clinically relevant. Urine cytology is widely used in the diagnostic evaluation of UTUC but has limited sensitivity, particularly for low-grade tumors [2]. In the present study, miR-191 and miR-200a were frequently detectable in patients with negative/suspicious cytology, suggesting that these miRNAs may provide information complementary to conventional cytology. However, this observation should not be interpreted as establishing UTUC-specific diagnostic performance. The control group consisted only of healthy individuals, and patients with other urothelial malignancies or benign urinary tract conditions were not included. Consequently, the present data demonstrate discrimination between patients with UTUC and healthy controls rather than the ability to distinguish UTUC from clinically relevant alternative diagnoses. Validation in appropriately selected disease-control cohorts will therefore be necessary to determine whether these miRNAs provide clinically useful diagnostic information beyond conventional cytology.

In contrast to miR-191 and miR-200a, miR-130b and miR-182 were detectable in only a small proportion of patients with UTUC and showed limited discriminatory performance. The low detection rates resulted in a large proportion of samples being assigned the upper Ct limit of 45, thereby limiting the interpretation of quantitative analyses involving these miRNAs. Although miR-182 detectability was significantly associated with pathological tumor stage and was observed only in patients with ≥pT2 disease, this finding should be interpreted cautiously because miR-182 was detectable in only eight patients with UTUC. miR-191 detectability was also more frequent in patients with ≥pT2 disease. Previous studies in bladder cancer have reported associations between miR-182 expression and tumor stage and grade [24]. Nevertheless, given the low detectability of miR-182 in the present cohort, its association with pathological stage should be regarded as exploratory and requires validation in larger cohorts.

Postoperative urinary miR-130b detectability showed an exploratory association with RFS. Although patients with detectable postoperative miR-130b had shorter RFS in the Kaplan–Meier analysis, the association did not reach statistical significance in either univariate or multivariable Cox regression analysis. Experimental studies in bladder cancer have demonstrated that miR-130b is upregulated in tumor tissues and cells and may promote proliferation, migration, and invasion through regulation of VGLL4 [25], providing a possible biological context for the present observation. However, given the small number of patients with detectable postoperative miR-130b and the limited number of recurrence events, the present finding cannot be considered evidence of independent prognostic significance. Whether postoperative urinary miR-130b reflects residual tumor-related biological activity or recurrence risk remains speculative and should be investigated in larger independent cohorts.

This study has several strengths, including its prospective multicenter design, standardized collection of urine samples within the JCOG1403A1 study, and availability of both preoperative and postoperative samples. In addition, urinary miRNAs were evaluated alongside conventional urine cytology, and a cell-sorting approach was used to select urinary cell fractions by excluding hematological cells before miRNA analysis. Several limitations should also be acknowledged. First, the sample size was relatively small, and the number of recurrence events and the numbers of patients with detectable miR-130b and miR-182 were limited. Second, the candidate miRNAs were selected from a small exploratory cohort, and neither the candidate miRNAs nor the combined miR-191/miR-200a model were validated in an independent cohort. Moreover, the combined model was developed and evaluated in the same relatively small cohort, including only 16 healthy controls, and its apparent discriminatory performance may therefore be subject to statistical optimism. Although bootstrap resampling was used to estimate uncertainty in the AUC, separate optimism-corrected internal validation was not performed. Further validation in an independent cohort is therefore warranted. Third, the control group consisted only of healthy individuals and did not include patients with bladder cancer or benign urinary tract conditions, precluding assessment of UTUC-specific diagnostic performance. In addition, the healthy control group was younger and consisted exclusively of men, resulting in substantial age and sex imbalances between the groups. Although the differences in miR-191 and miR-200a remained significant in a sensitivity analysis restricted to male participants and neither miRNA was significantly correlated with age within the UTUC cohort, residual confounding related to the substantial differences in age and sex distribution between the groups cannot be excluded. Finally, because the sorting strategy was based on physical properties without the use of a urothelial-specific marker, the cellular composition and purity of the FACS-selected urinary cell fraction could not be definitively established. The isolated fraction may therefore have contained heterogeneous urinary cells in addition to urothelial cells. In addition, because the urinary miRNAs measured in this study may originate from both the FACS-selected urothelial cell fraction and the associated fluid fraction, an appropriate endogenous reference for normalisation could not be established. We therefore analyzed target miRNAs using raw Ct values without normalisation to an endogenous internal reference, consistent with our previously reported approach. Nevertheless, this strategy cannot completely eliminate potential variability arising from RNA extraction and reverse transcription. Future validation using digital PCR, which enables direct absolute quantification without reliance on an endogenous reference, would further strengthen the analytical robustness of this approach.

In conclusion, urinary miR-191 and miR-200a showed potential for discriminating patients with UTUC from healthy controls and may complement urine cytology, particularly in patients with negative/suspicious cytology. However, their diagnostic specificity for UTUC remains to be established in comparisons with other urothelial malignancies and benign urinary tract conditions. The association between postoperative urinary miR-130b detectability and RFS should be considered exploratory and requires further validation. Larger studies with appropriately matched and clinically relevant control groups are warranted to clarify the potential clinical utility of urinary miRNAs in UTUC.

## Figures and Tables

**Table 1:** Patient characteristics.

	UTUC Patients	Healthy Controls
Number	45	16
Age, years, median (range)	71 (37–83)	47 (29–65)
Sex		
Male, *n* (%)	31 (69)	16 (100)
Female, *n* (%)	14 (31)	0 (0)
Urine cytology		
Positive, *n* (%)	14 (31)	
Negative/suspicious, *n* (%)	28 (62)	
Unknown/not assessed, *n* (%)	3 (7)	
Pathological T stage		
<pT2, *n* (%)	20 (44)	
≥pT2, *n* (%)	25 (56)	
Tumor grade		
High-grade, *n* (%)	35 (78)	
Low-grade, *n* (%)	10 (22)	
Lymphovascular invasion		
Present, *n* (%)	8 (18)	
Absent, *n* (%)	25 (56)	
Unknown, *n* (%)	12 (27)	
Divergent differentiation		
Present, *n* (%)	5 (11)	
Squamous differentiation, *n* (%)	5 (11)	
Glandular differentiation, *n* (%)	1 (2)	
Absent, *n* (%)	40 (89)	
Concomitant CIS		
Present, *n* (%)	18 (40)	
Absent, *n* (%)	27 (60)	
Tumor side		
Right, *n* (%)	20 (44)	
Left, *n* (%)	25 (56)	
Tumor location		
Renal pelvis, *n* (%)	24 (53)	
Ureter, *n* (%)	20 (44)	
Both renal pelvis and ureter, *n* (%)	1 (2)	
Multifocality		
Unifocal, *n* (%)	42 (93)	
Multifocal, *n* (%)	3 (7)	

Percentages for urine cytology were calculated using all 45 patients as the denominator; cytology was evaluable in 42 patients. One patient showed both squamous and glandular differentiation. Abbreviations: CIS, carcinoma in situ.

**Table 2:** Discriminatory performance of preoperative urinary sorting cell counts and candidate miRNAs for distinguishing patients with UTUC from healthy controls.

Biomarker	Cutoff	AUC	Sensitivity (%)	Specificity (%)
Sorting cell count	230.00	0.847	82.2	81.3
miR-130b	43.67	0.611	22.2	100.0
miR-182	42.23	0.562	15.6	100.0
miR-191	40.25	0.790	66.7	87.5
miR-200a	44.88	0.874	77.8	93.8
miR-191 + miR-200a	-	0.888	73.3	93.8

Cutoff values were determined using the Youden index from ROC analysis. For miRNAs, cutoff values represent Ct values, with lower Ct values indicating higher miRNA expression levels. No single Ct cutoff value was applicable to the combined miR-191/miR-200a model because the ROC analysis was based on the predicted probability derived from logistic regression.

**Table 3:** Detection rates of urine cytology and candidate urinary miRNAs in patients with UTUC.

Diagnostic Modality	Positive, *n* (%)
Urine cytology	14/42 (33.3)
miR-191	32/45 (71.1)
miR-200a	36/45 (80.0)
miR-191 or miR-200a	39/45 (86.7)
Cytology or miR-191 or miR-200a	39/42 (92.9)

Three patients had unavailable urine cytology results.

**Table 4:** Association between urinary miRNA detectability and pathological tumor stage.

miRNA	<pT2 (*n* = 20) Detectable, *n* (%)	≥pT2 (*n* = 25) Detectable, *n* (%)	*p* Value
miR-130b	4 (20.0)	6 (24.0)	1.0000
miR-182	0 (0.0)	8 (32.0)	0.0056
miR-191	11 (55.0)	21 (84.0)	0.0488
miR-200a	15 (75.0)	21 (84.0)	0.4817

Detectability was defined as Ct < 45. *p* values were calculated using Fisher’s exact test.

**Table 5:** Univariate and multivariate Cox proportional hazards analyses for RFS.

Variables	Univariate		Multivariate	
	HR (95% CI)	*p* Value	HR (95% CI)	*p* Value
Age	0.98 (0.94–1.03)	0.326		
Female sex	1.37 (0.46–4.27)	0.558		
Positive urine cytology	1.25 (0.41–3.82)	0.697		
≥pT2	1.18 (0.38–3.61)	0.771		
High-grade tumor	0.39 (0.12–1.27)	0.118		
LVI positive	0.79 (0.22–2.86)	0.721		
Concomitant CIS	1.65 (0.55–4.93)	0.366		
No postoperative intravesical pirarubicin instillation	3.89 (0.99–15.22)	0.051	3.59 (1.09–11.84)	0.036
Postoperative miR-130b detectability	3.14 (0.96–10.25)	0.058	2.76 (0.84–9.05)	0.094

Multivariate analysis included postoperative intravesical pirarubicin instillation and postoperative miR-130b detectability. Abbreviation: RFS, relapse-free survival; HR, hazard ratio; CI, confidence interval.

## Data Availability

The data presented in this study are available on request from the corresponding author, subject to approval by the investigators from the Urologic Oncology Study Group of JCOG. The data are not publicly available due to ethical and institutional restrictions.

## Supplementary Materials

The following supporting information can be downloaded at https://www.mdpi.com/article/10.3390/genes17091145/s1, Figure S1: Correlation between preoperative urinary sorting cell counts and candidate miRNA Ct values in patients with UTUC.; Figure S2: Relapse-free survival according to postoperative urinary miRNA detectability.; Table S1: Candidate miRNAs selected for validation analysis.; Table S2: Ranking of candidate miRNAs in the exploratory sequencing analysis.; Table S3: Relationship between urine cytology and combined detectability of miR-191 and miR-200a in patients with UTUC.; Table S4: Univariate Cox proportional hazards analysis of postoperative urinary miRNA detectability for relapse-free survival in patients with UTUC.; Table S5: Multivariable logistic regression analysis of miR-191 and miR-200a for discrimination between patients with UTUC and healthy controls after adjustment for sorted cell count; Table S6: Sensitivity analyses assessing the potential effects of sex and age on urinary miR-191 and miR-200a levels; Table S7: Discriminatory performance of urinary miR-191, miR-200a, and the combined model in the overall cohort.

## Abbreviations

The following abbreviations are used in this manuscript:

JCOG	Japan Clinical Oncology Group
miRNA	microRNA
UTUC	Upper tract urothelial carcinoma
RFS	Relapse-free survival
ROC	Receiver operating characteristic
AUC	Area under the curve
HR	Hazard ratio
CI	Confidence interval
