# Supplementary material for: Evaluation of Candidate Urinary microRNAs in Upper Tract Urothelial Carcinoma: A Prospective Multicenter Biomarker Study (JCOG1403A1)
Source: Genes (Basel). 2026 Sep 18;17(9):1145. doi: 10.3390/genes17091145 (PMC13606361; doi:10.3390/genes17091145)
Supplement: Supplementary file 1 [file genes-17-01145-s001.zip › genes-4570413-supplementary.pdf]

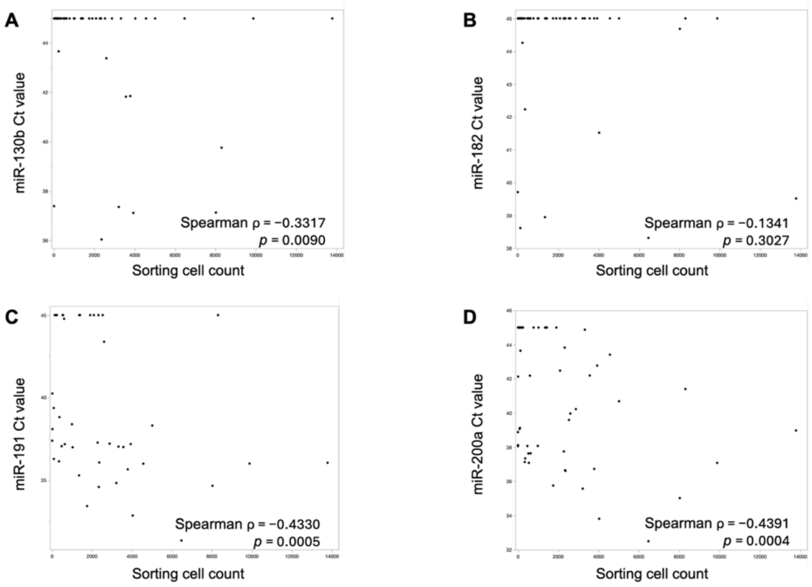

Supplementary Figure S1. Correlation between preoperative urinary sorting cell counts and candidate miRNA Ct values in patients with UTUC. Scatter plots showing the relationship between urinary sorting cell counts and expression levels (Ct values) of (A) miR-130b, (B) miR-182, (C) miR-191, and (D) miR-200a. Correlations were evaluated using Spearman's rank correlation coefficient. A Ct value of 45 was assigned to samples with no detectable amplification.

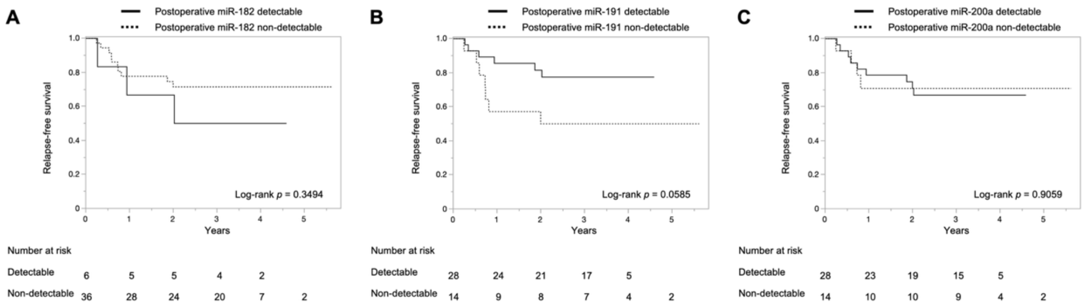

Supplementary Figure S2. Relapse-free survival according to postoperative urinary miRNA detectability. Kaplan–Meier curve for relapse-free survival stratified by postoperative detectability of (A) miR-182, (B) miR-191, and (C) miR-200a. Detectability was defined as Ct < 45. P values were calculated using the log-rank test.

Supplementary Table S1. Candidate miRNAs selected for prospective validation.

miRNAs	Rank	Previous evidence in urothelial carcinoma	Rational for selection
miR-130b	1	Li Q, et al., Mol Diagn Ther. 2019. [5]<br>Li B, et al., Arch Med Sci. 2019. [6]	High ranking in the exploratory analysis and previous evidence in urothelial carcinoma
miR-182	2	Zhang QH, et al, Gene. 2013. [7].	High ranking in the exploratory analysis and previous evidence in urothelial carcinoma
miR-191	11	Tao J, et al., Mol Med Rep. 2015. [14]<br>Ozden, SB et al., BMC Urol. 2026. [8]	Exploratory sequencing results and previous evidence in urothelial carcinoma
miR-200a	33	Cinque A, et al., Int J Mol Sci. 2022. [15]<br>Wan P, et al., Trans Androl Urol. 2021. [9]	Exploratory sequencing results and previous evidence in urothelial carcinoma

Candidate miRNAs were selected by considering both the results of the exploratory sequencing analysis and previous evidence regarding their potential relevance to urothelial carcinoma. Selection was not based solely on the expression-score ranking. Detailed results of the exploratory sequencing analysis, including the expression score and its components, are provided in Supplementary Table S2A.

Supplementary Table S2. Part A. Group-level expression data and calculation of the expression score in the exploratory sequencing analysis.

miRNAs	Mean Normal	SD Normal	Mean Cancer	SD Cancer	Difference Cancer − Normal	LogFC	*p* value	Score	Rank
miR-130b-3p	6.55	1.70	78.73	27.68	72.19	4.436	0.0012	9344.87	1
miR-182-5p	434.82	467.94	8053.51	1768.60	7618.69	4.679	0.0019	8286.64	2
miR-20a-5p	33.36	16.53	2752.13	3383.65	2718.77	6.562	0.0010	5074.24	3
miR-17-5p	36.90	2.05	705.38	632.66	668.49	4.603	0.0017	2935.00	4
miR-183-5p	169.99	106.37	1220.54	203.58	1050.55	3.419	0.0090	1294.34	5
miR-3607-3p	5.25	2.11	91.39	112.85	86.14	4.582	0.0044	772.35	6
miR-16-5p	820.42	618.14	13466.65	6590.12	12646.23	4.188	0.0105	697.02	7
miR-421	9.53	1.66	58.24	15.95	48.71	3.147	0.0157	553.46	8
miR-542-3p	5.73	6.94	60.71	20.96	54.97	3.600	0.0135	524.84	9
miR-34a-5p	70.63	0.74	590.22	494.14	519.59	3.487	0.0077	474.81	10
miR-191-5p	2399.88	1441.52	17912.47	8563.05	15512.59	3.448	0.0144	370.14	11
miR-98-5p	174.98	167.38	1849.93	1170.73	1674.94	3.489	0.0129	338.52	12
miR-15a-5p	44.13	52.65	637.78	360.12	593.65	3.857	0.0206	268.77	13
miR-146-5p	427.09	190.82	2165.45	787.20	1738.36	2.943	0.0215	243.00	14
miR-215-5p	13.12	15.64	242.29	288.94	229.17	4.264	0.0133	240.86	15
miR-30b-5p	662.26	220.42	5608.00	6273.11	4945.75	3.409	0.0162	160.74	16
miR-425-5p	101.68	12.28	447.36	189.14	345.68	2.598	0.0431	103.53	17
miR-126-3p	220.13	254.96	2323.07	2438.44	2102.94	3.422	0.0290	91.98	18
miR-301a-3p	31.64	41.54	255.49	105.58	223.85	3.072	0.0612	76.36	19
miR-126-5p	1826.83	1571.54	13262.14	13270.35	11435.31	3.036	0.0308	75.89	20
miR-96-5p	10.06	12.50	105.21	124.26	95.15	3.464	0.0336	71.71	21
miR-192-5p	1465.80	907.95	12452.97	16265.06	10987.18	3.478	0.0341	65.23	22
miR-93-5p	285.59	127.75	1305.73	637.69	1020.14	2.661	0.0549	64.56	23
miR-151-3p	1667.12	471.84	4617.99	1423.03	2950.87	2.227	0.0661	52.43	24
miR-30c-5p	774.30	40.12	3100.75	1751.48	2326.45	2.364	0.0587	52.30	25
miR-190-5p	10.26	11.95	84.07	81.82	73.81	3.111	0.0498	49.15	26
miR-15b-5p	54.61	45.12	276.26	206.42	221.65	2.803	0.0570	43.36	27
miR-193a3p	43.81	3.30	141.78	103.92	97.97	2.260	0.0530	38.97	28
miR-582-3p	7.62	7.72	62.42	77.32	54.81	2.999	0.0514	37.62	29
miR-141-3p	719.62	867.74	6855.94	7312.44	6136.31	3.289	0.0710	34.77	30
miR-138-5p	55.28	7.16	257.47	308.80	202.19	2.923	0.0544	34.36	31

miR-195-5p	109.69	88.69	684.98	640.13	575.29	2.783	0.0652	33.70	32
miR-200a-3p	97.82	91.61	613.96	590.92	516.14	2.740	0.0641	32.34	33
miR-196b-5p	81.40	22.63	312.99	136.15	231.60	2.443	0.1165	30.58	34
miR-335-5p	9.19	11.42	51.09	29.41	41.90	2.499	0.0868	29.53	35
miR-107	253.67	140.24	842.69	370.82	589.01	2.073	0.0821	29.10	36
miR-455-5p	31.29	19.66	112.19	59.14	80.90	2.154	0.0805	27.47	37
miR-151a-5p	479.67	97.78	2011.49	1485.71	1531.82	2.438	0.0895	26.36	38
miR-204-5p	556.57	114.10	2451.70	3239.92	1895.13	2.729	0.0625	24.69	39
miR-148a-3p	2946.43	2212.38	9168.18	4815.15	6221.76	2.250	0.0974	20.45	40

Expression values are presented as the mean and population standard deviation (SD; calculated using *n* as the denominator) for the normal and cancer groups. Difference was calculated as Mean Cancer − Mean Normal. The expression score was calculated as follows: Score = (Mean Cancer − Mean Normal) × logFC / (SD Cancer + SD Normal) / *p* value. miRNAs were ranked in descending order according to the calculated score. Calculations were performed using unrounded values; displayed values were rounded for presentation. Individual sample-level expression values are provided in Supplementary Table S2B. Abbreviations: FC, fold change; SD, standard deviation.

Supplementary Table S2. Part B. Individual sample-level expression data from the exploratory sequencing analysis.

miRNAs	Normal 1	Normal 2	Normal 3	Cancer 1	Cancer 2	Cancer 3
miR-130b-3p	2.40	5.79	11.46	104.61	91.23	40.36
miR-182-5p	86.89	1022.78	194.79	10431.25	7536.68	6192.59
miR-20a-5p	33.41	66.47	0.21	141.11	584.81	7530.47
miR-17-5p	47.89	51.98	10.81	84.29	458.19	1573.67
miR-183-5p	32.46	245.20	232.32	1118.20	1504.76	1038.67
miR-3607-3p	9.49	5.27	1.00	6.99	16.29	250.90
miR-16-5p	608.44	1844.73	8.09	4207.56	17176.16	19016.24
miR-421	15.28	11.95	1.36	39.42	78.42	56.88
miR-542-3p	0.55	14.43	2.22	31.31	72.07	78.74
miR-34a-5p	85.19	83.72	42.97	96.98	408.12	1265.56
miR-191-5p	1903.13	4786.18	510.32	24267.53	23662.34	5807.53
miR-98-5p	44.39	379.15	101.41	294.34	3118.67	2136.78
miR-15a-5p	13.33	118.63	0.43	128.83	876.38	908.15
miR-146-5p	65.97	447.61	767.70	1256.95	2062.46	3176.94
miR-215-5p	2.25	33.52	3.58	7.76	69.77	649.34
miR-30b-5p	581.83	1022.66	382.28	570.64	1802.41	14450.97
miR-425-5p	131.94	156.50	16.61	330.40	714.17	297.51
miR-126-3p	58.63	568.54	33.23	951.91	5748.90	268.41
miR-301a-3p	5.89	88.96	0.07	108.07	349.74	308.65
miR-126-5p	302.42	3445.50	1732.56	4643.41	32009.01	3134.01
miR-96-5p	1.55	26.55	2.08	12.01	22.78	280.83
miR-192-5p	107.57	1923.47	2366.35	530.70	1378.18	35450.03
miR-93-5p	551.16	295.66	9.95	406.12	1810.23	1700.84
miR-151-3p	1429.67	2373.36	1198.32	6627.48	3518.36	3708.13
miR-30c-5p	1019.18	1099.42	204.32	823.03	3396.70	5082.52
miR-190-5p	0.75	24.65	5.37	3.43	52.52	196.26
miR-15b-5p	126.39	36.14	1.29	29.48	534.71	264.60
miR-193a3p	33.21	26.61	71.61	44.16	285.74	95.46
miR-582-3p	2.60	18.03	2.22	8.85	171.76	6.66
miR-141-3p	199.20	1934.68	24.99	3553.21	16994.15	20.45
miR-138-5p	5.44	19.76	140.65	21.09	57.66	693.67
miR-195-5p	73.41	250.79	4.87	347.67	1581.18	126.09
miR-200a-3p	50.89	234.12	8.45	275.49	1444.90	121.50

miR-196b-5p	99.43	144.69	0.07	268.72	172.85	497.41
miR-335-5p	1.65	24.48	1.43	9.56	69.68	74.02
miR-107	129.99	410.46	220.57	319.13	1078.45	1130.48
miR-455-5p	16.78	56.10	20.98	40.14	111.44	185.00
miR-151a-5p	809.44	613.87	15.68	290.72	1827.73	3916.02
miR-204-5p	604.60	376.39	688.71	131.91	189.68	7033.51
miR-148a-3p	1729.45	6154.21	955.62	13355.00	2423.81	11725.74

Individual expression values are shown for the three normal and three cancer samples used in the exploratory sequencing analysis. These values were used to calculate the group means and population standard deviations presented in Supplementary Table S2.

Supplementary Table S3. Relationship between urine cytology and combined detectability of miR-191 and miR-200a in patients with UTUC.

	Cytology positive (n=14)	Cytology negative/suspicious (n =28)
miR-191 detectable	9 (64.2%)	22 (78.6%)
miR-200a detectable	10 (71.4%)	24 (85.7%)
Either miR-191 or miR-200a detectable	12 (85.7%)	25 (89.3%)
Neither miR-191 nor miR-200a detectable	2 (14.3%)	3 (10.7%)

Supplementary Table S4. Univariate Cox proportional hazards analysis of postoperative urinary miRNA detectability for relapse-free survival in patients with UTUC.

miRNA	Detectable, n	Non-detectable, n	HR	95% CI	*p* value
miR-130b	7	35	3.14	0.96–10.25	0.058
miR-182	6	36	1.83	0.51–6.67	0.357
miR-191	30	12	0.36	0.12–1.08	0.070
miR-200a	29	13	1.07	0.33–3.49	0.906

Hazard ratios were calculated using univariate Cox proportional hazards regression analysis. Detectability was defined as Ct < 45. Non-detectable samples were assigned a Ct value of 45.

Supplementary Table S5. Multivariable logistic regression analysis of miR-191 and miR-200a for discrimination between patients with UTUC and healthy controls after adjustment for sorted cell count.

Model	Variable	OR	95% CI	p value
Model 1	miR-191 Ct	0.598	0.373–0.809	0.008
	log10 (sorted cell count + 1)	4.926	1.956–18.649	0.005
Model 2	miR-200a Ct	0.433	0.204–0.684	0.005
	log10 (sorted cell count + 1)	4.759	1.748–20.014	0.01

Lower Ct values indicate higher miRNA abundance. Sorted cell count was log10-transformed as log10(sorted cell count + 1) because of its right-skewed distribution. Each model included the indicated miRNA Ct value and log10-transformed sorted cell count as covariates. ORs are reported per one-unit increase in each variable. Ct, cycle threshold; OR, odds ratio; CI, confidence interval; UTUC, upper tract urothelial carcinoma.

Supplementary Table S6. Sensitivity analyses assessing the potential effects of sex and age on urinary miR-191 and miR-200a levels.

Analysis	miRNA	n	Result	p value
Male-only comparison (UTUC vs healthy controls)	miR-191	31 vs 16	AUC, 0.770	0.0014†
	miR-200a	31 vs 16	AUC, 0.862	<0.0001†
Correlation with age in patients with UTUC	miR-191	45	ρ = -0.158	0.299
	miR-200a	45	ρ = -0.204	0.179

†P values were calculated using the Mann–Whitney U test. Correlations with age were assessed using Spearman's rank correlation coefficient. Lower Ct values indicate higher miRNA abundance. Abbreviations: AUC, area under the receiver operating characteristic curve; Ct, cycle threshold; UTUC, upper tract urothelial carcinoma.

Supplementary Table S7. Discriminatory performance of urinary miR-191, miR-200a, and the combined model in the overall cohort.

Marker/model	AUC (95% CI)	Exploratory cut-off	Sensitivity (95% CI)	Specificity (95% CI)
miR-191	0.790 (0.674–0.881)	Ct 40.245	66.7% (51.0–80.0)	87.5% (61.7–98.4)
miR-200a	0.874 (0.780–0.940)	Ct 44.876	77.8% (62.9–88.8)	93.8% (69.8–99.8)
miR-191 + miR-200a	0.888 (0.778–0.948)	Predicted Probability, 0.715	73.3% (58.1–85.4)	93.8% (69.8–99.8)

AUC, area under the receiver operating characteristic curve; CI, confidence interval; Ct, cycle threshold. AUCs are presented with 95% CIs estimated using bias-corrected bootstrap resampling with 5,000 replicates. Sensitivity and specificity are presented with exact binomial (Clopper–Pearson) 95% CIs. Cut-off values were determined using the Youden index and should be considered exploratory. The combined model was constructed using logistic regression incorporating miR-191 and miR-200a Ct values.
